# Health systems facilitators and barriers to the integration of HIV and chronic disease services: a systematic review

**DOI:** 10.1093/heapol/czw149

**Published:** 2017-11-24

**Authors:** Nicola Watt, Louise Sigfrid, Helena Legido-Quigley, Sue Hogarth, Will Maimaris, Laura Otero-García, Pablo Perel, Kent Buse, Martin McKee, Peter Piot, Dina Balabanova

**Affiliations:** 1 The Centre for Health and Social Change (ECOHOST), London School of Hygiene and Tropical Medicine, 15-17 Tavistock Place London, London WC1H 9SH, UK; 2 Centre for Tropical Medicine and Global Health, Nuffield Dept. of Medicine, University of Oxford, Oxford, UK; 3 Saw Swee Hock School of Public Health, National University of Singapore, 12 Science Drive 2, #10-01, Tahir Foundation Building, 117549 Singapore; 4 London School of Hygiene and Tropical Medicine, 15-17 Tavistock Place, London WC1H 9SH, UK; 5 Public Health Consultant at Tower Hamlets Together; 6 Public Health Consultant, Haringey Council, London; 7 Nursing Section, Faculty of Medicine, Universidad Autonoma de Madrid, Arzobispo Morcillo Av., 4, Madrid and CIBER of Epidemiology and Public 15 Health (CIBERESP), Madrid, Spain; 8 Chief, Strategic Policy Directions, UNAIDS, Geneva, Switzerland; 9 Department of Global Health and Development, London School of Hygiene and Tropical Medicine, 15-17 Tavistock Place, London WC1H 9SH, UK

**Keywords:** Barriers to access, chronic disease, health care delivery, health system, HIV, integrated care, integration

## Abstract

Integration of services for patients with more than one diagnosed condition has intuitive appeal but it has been argued that the empirical evidence to support it is limited. We report the findings of a systematic review that sought to identify health system factors, extrinsic to the integration process, which either facilitated or hindered the integration of services for two common disorders, HIV and chronic non-communicable diseases. Findings were initially extracted and organized around a health system framework, followed by a thematic cross-cutting analysis and validation steps. Of the 150 articles included, 67% (*n* = 102) were from high-income countries. The articles explored integration with services for one or several chronic disorders, the most studied being alcohol or substance use disorders (47.7%), and mental health issues (29.5%). Four cross-cutting themes related to the health system were identified. The first and most common theme was the requirement for effective collaboration and coordination: formal and informal productive relationships throughout the system between providers and within teams, and between staff and patients. The second was the need for adequate and appropriately skilled and incentivized health workers—with the right expertise, training and operational support for the programme. The third was the need for supportive institutional structures and dedicated resources. The fourth was leadership in terms of political will, effective managerial oversight and organizational culture, indicating that actual implementation is as important as programme design. A fifth theme, outside the health system, but underpinning all aspects of the system operation, was that placing the patient at the centre of service delivery and responding holistically to their diverse needs. This was an important facilitator of integration. These findings confirm that integration processes in service delivery depend substantially for their success on characteristics of the health systems in which they are embedded.

Key Messages

- Effective collaboration and coordination, comprising formal and informal productive relationships throughout the health system, and between the health system, patients and communities, trained and incentivized health workers (including dedicated coordinators) with access to clear guidelines, were essential facilitators of integration.
- Accessible and acceptable physical and institutional structures are important, but the need for patient-centred delivery taking into account patients’ complex socio-economic and cultural needs was identified as a strong theme in itself.
- Leadership (including political will to implement integration, defining integration as an explicit goal and having a vision and clearly defined strategy for implementing it) and supportive organizational culture emerged as essential underlying characteristics.
- Service delivery integration imposes transaction costs; communication and collaboration—important facilitators for integration—are not cost free.

## Introduction

The past two decades have seen an unprecedented increase in investment in health worldwide. Encouraged by evidence about the role of poor health as a brake on economic growth,(World Health Organization 2001) the scale and nature of the global burden of disease (Murray and Lopez 1996), and new opportunities to intervene, including advances in medicines and vaccines, and with a sense of urgency created by the emergence of HIV, countries have placed health high on the global political agenda. First in the Millennium Development Goals (Bhutta *et al.* 2010), and now in the Sustainable Development Goals, the international community has committed to adopt measures that will reduce substantially the toll of disease and premature death, especially among the poor, living in countries at all levels of economic development, who suffer most (Marmot *et al.* 2008). The scope of these measures has extended progressively from an initial focus on maternal and child health to encompass the growing threat from non-communicable diseases (NCDs) and the importance of universal health coverage as a prerequisite for sustained progress (Vega 2013; WHO 2013).

Over most of this period the increased investment was channelled through a growing number of global health initiatives, each targeted on specific diseases, such as the Global Fund to Fight AIDS, Tuberculosis and Malaria, or on particular technologies, such as Gavi, with its focus on vaccines. These, in turn, supported many largely vertical, and sometimes fragmented programmes to implement the goals of each funder. However, while considerable progress on selected health issues has been made, there is now widespread recognition that much more is possible by creating synergies across programmes with the available resources (Balabanova *et al.* 2010).

In response to these concerns, there have been growing calls from researchers and policy makers for greater integration, both at the policy level, bringing together programmes that each address a single health problem, and at the operational level, ensuring that scarce resources are used as efficiently as possible (WHO 2008a,b). At service delivery level, integration can manifest in many different ways (Box 1). Integration can be between disease-specific (vertical) programmes and system-wide (horizontal) structures and policies (WHO 2008a,b), across related disease programmes (e.g. maternal and reproductive care), between public health and health service interventions, and between health systems and other sectors. This article focuses on integration of service delivery for diseases that are usually delivered separately but often affect the same types of end-users.

The proposition underlying the development of integrated programmes and interventions is that they can offer the means to improve access and responsiveness to patients’ needs, increase coverage, reduce inequalities, and improve health outcomes (Atun *et al.* 2010b). Integration is seen as particularly promising as a way of introducing effective and feasible delivery models in high burden and low resource settings: it may help to ensure that multiple health needs are addressed, build on commonalities in care delivery and drug distribution systems, enable sharing of facilities and other capital resources and align funding mechanisms (Brady *et al.* 2006). For these reasons, integration of separate initiatives within health systems is increasingly seen as a precondition for sustainability and is high on national and international health agendas (Shigayeva and Coker 2014).

**Figure 1.**
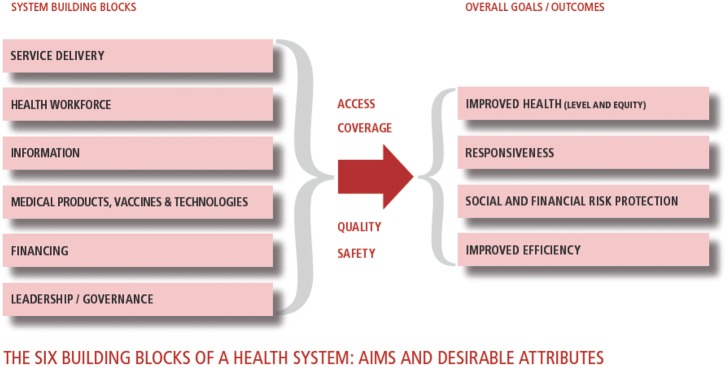
Health systems ‘building block’ framework. Source: (WHO 2007)

Yet although the arguments in favour of integration have an intuitive appeal to policymakers, the evidence that it achieves real benefits is less clear (Shigayeva *et al.* 2010). Much seems to come down to how programmes are implemented. Several studies have highlighted the importance of achieving a shared understanding of the rationale for integration among those delivering previously separate programmes, as well as adequate investment in a range of new resources and in training. Thus, despite a strong theoretical case for integrating services for HIV and maternal and child health in Swaziland, access was reduced (Church *et al.* 2013). Even where there is institutional support, service integration is not a quick fix to improve quality and accessibility of care, and similarly, even where services operate side by side, genuine implementation can be constrained by competing management priorities and social norms shaping the interaction between providers and population (Mounier-Jack *et al.* 2014). Previous research on integration is typically focused on endogenous factors, related to the characteristics of the programmes involved, such as health workers, medicines and knowledge. Rather less attention has been given to exogenous factors, and particularly those that reside outside the particular programme or relate to the broader health system. This is despite a growing recognition of the importance of ensuring that a series of building blocks are in place in any health system to enhance its ability to deliver effective care (WHO 2007) (Figure 1).

Health systems building blocks are interdependent; interventions in one block will have intended or unintended consequences for, or dependencies on, other blocks. For example, an electronic patient record system may only facilitate integration if staff have been adequately trained in its use and are motivated to apply it in their daily practice. Thus, interventions are needed both in the areas of improving information collection and use and in workforce policies. Moreover, the oversight of the building blocks requires an effective governance framework, including monitoring and evaluation and incorporating lessons learnt, and in a health system that has the means to generate continually the resources needed to support investment in structures and processes (Adam and De Savigny 2012).

This article reviews what is known about facilitators of and barriers to integration of health care for two sets of conditions that have much in common yet are often delivered separately. These are HIV and other long-term conditions. The latter includes both chronic diseases, defined as prolonged, often lifelong conditions that ‘persist and require some level of health care management across time’ (World Health Organization 2002), such as diabetes and other NCDs, as well as damaging health behaviours for which long-term treatment is offered, such as substance use disorders (for brevity we use this term throughout this article, recognizing that terminology varies and includes a spectrum of conditions, as reflected in the search strategy). Since long term treatment with anti-retrovirals now both allows those living with HIV to survive into old age, when they are at greater risk of common NCDs, and increases their risk of acquiring some of these conditions, such as cardiovascular disease (Nigatu 2012; Deeks *et al.* 2013; Post *et al.* 2014), infection with HIV is increasingly being viewed as a chronic disorder (Deeks *et al.* 2013). HIV and other long-term conditions involve long term treatment and continuous follow-up, with input from health workers with different knowledge and skills, community and family support and the active participation of the patient (Nigatu 2012; Haregu *et al.* 2015). Successful treatment depends on an understanding of the nature of what, in many cases, are asymptomatic conditions, and the importance of long term adherence to treatment. Despite differing aetiologies therefore, there is a growing understanding of the medical and organizational arguments for integrating HIV and other chronic disorders (UNAIDS 2011). This article is part of a series of related articles commissioned by UNAIDS that draw on the same review but explore different combinations of services and delivery configurations and their outcomes.

## Methods

### Objective

To identify barriers and facilitators at the level of the health system which influence the success or failure of measures to integrate programmes for HIV and chronic conditions.

### Definitions

Drawing on the definitions proposed by Briggs and Garner (2006), Atun *et al.* (2010b) and Legido-Quigley *et al.* (2013) we define integration as managerial or operational changes to health systems to bring together inputs, delivery, management and organization of particular service functions as a means of improving coverage, access, quality, acceptability and (cost)-effectiveness. We consider this to include integration of supply chains, interventions that combine ‘different packages of services’; the integration of service delivery points; integration at different levels of service delivery; process modifications; the introduction of technologies aimed at aiding integration; and integration of management decisions (WHO 2008b, accessed 20 May 2010. Available from: http://www.who.int/healthsystems/technical_brief_final.pdf, Atun *et al.* 2010a).

A detailed protocol was developed and is reproduced in the Supplementary Materials. Health systems barriers and facilitators were located within the WHO health systems concept, with systems defined as ‘all the organizations, institutions and resources that are devoted to producing health actions’, and comprising the ‘building blocks’ mentioned above (WHO 2007). To be considered for inclusion, the study must describe efforts which sought to integrate care of a chronic condition with that for HIV (see Supplementary Table S1, for a full list of chronic conditions included in the review).

### Eligibility criteria

To ensure inclusion of as wide a range of articles, and findings, as possible, there were no restrictions on language or study design because we recognized that all types of methods could shed light on barriers or facilitators. We included all quantitative, qualitative and mixed method studies that reported primary research findings on health system policies, interventions or programmes in relation to different models of integrated HIV and services for chronic conditions. Purely descriptive studies or commentaries were excluded, however reviews which presented primary study findings not reported elsewhere were included. In summary, in order to be included, the studies had to have the following characteristics:

- A description or evaluation of a management or organisational change strategy, implemented within an existing health system, aiming to increase integration of services delivering care for people with HIV and other chronic conditions.
- Actual experience of integration, rather than just a theoretical account of how integration might be implemented.
- Screening or treatment for HIV in a service where the focus was on other chronic conditions or vice versa (e.g. interventions related to HIV in the course of screening for other conditions)
- Services that went beyond simply diagnostic/screening procedures to include some therapeutic intervention (which could range from counselling to invasive procedures)

These included services provided in health facilities or the community. However, studies of integration of HIV services with TB services (Legido-Quigley *et al.* 2013), maternal or family planning services (De Jongh *et al.* 2016), were excluded as they have been described in recent reviews.

### Search strategy

The search strategy was designed collaboratively with an information specialist to be consistent with methods used by other authors for systematic reviews of integration of health services (Briggs and Garner 2006; Atun *et al.* 2010a). The following electronic databases were searched from inception until October 2015: Global Health, Medline and Embase. In addition, the following databases were searched using a simplified search strategy to ensure that articles from low- and middle-income countries (LMICs) were found: Cochrane library, Latin American and Caribbean Health Sciences Literature(LILACs), Africa Wide, WHOLIS and abstracts from the International AIDS Society Online Resource Library from 2006 to 2015, the HIV Implementers meetings from 2007 to 2012 and International conferences on NCDs such as the 2014 Annual Meeting of the College on Problems of Drug Dependence or the 2015 Annual Scientific Meeting of the Research Society on Alcoholism among others. Key words MeSH (Medical Subject Headings) and free text terms developed for three themes: HIV, integration and chronic conditions and then combined in the search strategy (see protocol in Supplementary Materials)].

### Analytical approach

**Figure 2.**
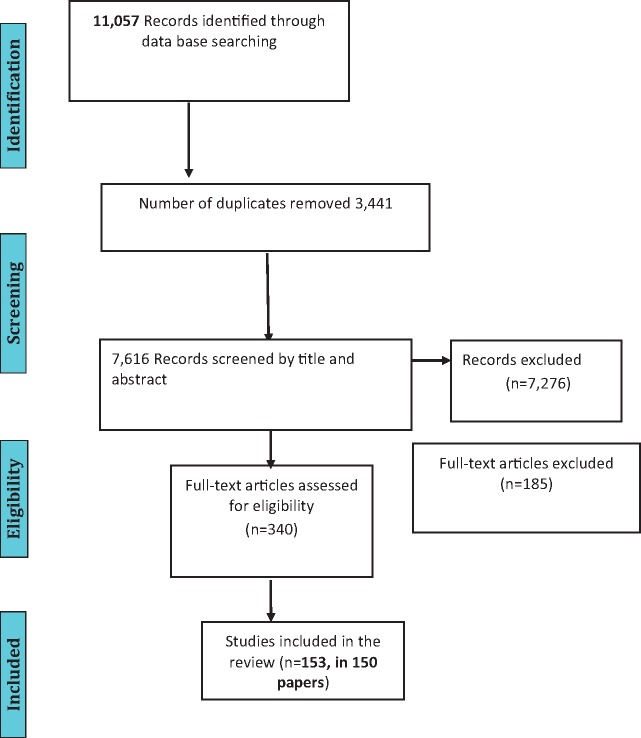
Study flow diagram.

The protocol was designed for extracting data on study design, setting, health system domains, research methods, outcomes and risk of bias assessments and was operationalized by creating extraction forms for both quantitative and qualitative studies. The same protocol was applied to all studies, with the only differences in data extraction and analysis being in the assessment of risk of bias for qualitative versus quantitative studies. These forms were piloted by the reviewers on two randomly selected studies included in the review and feedback was then incorporated in a revision. Two reviewers independently reviewed the list of articles from the electronic search results to identify relevant articles based on title or title and abstract. They independently assessed whether the retrieved full texts met the inclusion criteria, with those failing to do so excluded (Figure 2). Any disagreements were resolved through discussion with a third reviewer. Studies which present evaluative rather than purely descriptive data were independently assessed for risk of bias by two reviewers (see Supplementary Table S3 and Box A1).

At the first stage, two reviewers independently coded barriers and enablers of integration according to the health system building block(s) to which they related, ensuring that data extraction sufficiently captures the context. The findings in both quantitative and qualitative studies were coded in a similar way. The themes that emerged were then compared and discussed by the two reviewers to ensure coherence in their coding. This process showed that the building blocks, while useful for an initial categorization, were limited because interventions often manifested in more than one block, or interventions in one block dependent on actions under a different block. Thus, at the second stage, three reviewers identified the cross-cutting themes emerging across (more than one) health system blocks. Subthemes under each main cross-cutting theme were agreed by at least two reviewers by exploring at results across the relevant blocks. At the third stage, after identifying a series of cross cutting facilitators and barriers, two reviewers independently reviewed the results in each theme to identify divergent cases. All three reviewers discussed and adjusted the final overarching themes and agreed on an assessment of the level of generalizability, based on the numbers of articles identifying a particular theme and the strength of the evidence within those articles.

## Results

### Overview of studies included

Key characteristics of the 153 studies in the 150 articles that met the eligibility criteria are set out in the Supplementary Table S2. A majority of studies (67%, *n* = 102) were set in high-income countries as classified by the World Bank (2015) with 59% (*n* = 90) from North America. Study participants included people with confirmed HIV infection, people offered screening for HIV within the setting of other services, or health care providers. Some studies included whole populations from areas of high HIV prevalence or risk. A number of studies also included partners or family members of people living with HIV and some included health system directors or managers. The studies covered a wide range of chronic conditions, some involving integration of HIV and one additional chronic condition while some included a combination of several chronic conditions. Most commonly, studies were descriptive (43) including 22 cohort studies (including retrospective as well as prospective), 23 cross-sectional studies, 12 qualitative studies and 13 randomized controlled trials (see Supplementary Table S2). Risk of bias assessments were carried out for the 40 evaluative studies, of which 23 were graded as ‘high’, three as ‘moderate ’ and only two as ‘low’ (the rest were ‘unclear’).

### Health systems facilitators and barriers

Following the analytical process described earlier, the review findings were divided into key overarching themes that cut across the different dimensions (building blocks) of health systems. The key cross cutting themes on facilitators and barriers to integration are presented in Table 1. The themes pertaining to each individual health systems block (stage 1 of the analysis) are presented in the Supplementary Table S4.

**Table 1.** Cross-cutting themes

Cross-cutting theme	Subtheme	±	Generalizability	Illustrative references	Recommendations
**Collaboration/coordination/ relationships/links**	Strong relationships between providers and stakeholders	±	Strong: range of settings and programmes	(Dodds et al. 2004; UNAIDS, 2011; Odafe et al. 2013)	Programme design and staffing should allow sufficient opportunity to build formal and informal linkages and ensure patients well informed: named coordinators may be beneficial in certain circumstances and for complex needs
	Strong links, communication and collaboration between providers.	±	Strong: range of settings and programmes	(Feingold and Slammon 1993; Andersen et al. 2003; Bouis et al. 2007)	
	Coordination and case management of individual’s care—including coordination/navigation of care by an identified person (coordinator/advocate/nurse practitioner)	±	Moderate: Particularly for complex needs around mental health or substance abuse, high-income settings	(Feingold and Slammon 1993; Finkelstein et al. 2011)	
	Information sharing between staff/providers—including regulatory barriers to info sharing	±	Moderate: several articles,	(Lombard et al. 2009; Inouye et al. 2011; Mwanahamuntu et al. 2011)	
	Information for patients (including accounting for cultural issues)	±	Strong: range of settings and programmes	(Ibrahim et al. 2009; Odafe et al. 2013; Khozaim et al. 2014)	
**Health workers: trained, available, multidisciplinary, motivated, incentivized**	Availability of human resources including specialist staff	±	Strong: range of settings and programmes	(Wood, 2008; Egan et al. 2011, Edwards et al. 2015; Kumakech et al. 2015)	Resourcing should ensure adequate staff from the necessary disciplines, plus training and support as appropriate
	Staff education, training, expertise, skills and experience including ongoing support, supervision and training	±	Strong: range of settings and programmes	(Altice et al. 2004; Clanon et al. 2005; Ibrahim et al. 2009; Huchko et al. 2011; Kieran et al. 2011; Nyabera et al. 2011; Horo et al. 2012; Moon et al. 2012; Ramogola-Masire et al. 2012; Hasin et al. 2013; Odafe et al. 2013; Khozaim et al. 2014; Martin et al. 2014; Kotwani et al. 2014; Anderson et al. 2015)	
	Multidisciplinary teams	+	Strong: range of settings and programmes	(Wood, 2008; Edwards et al. 2015; Kumakech et al. 2015)	
	Staff culture, interest, awareness, enthusiasm—ie whether or not the staff are motivated and want to engage	±	Moderate: several articles, mostly US Substance Abuse	(Cheever et al. 2011; Curran et al. 2011)	
	Financial incentives to take part (adopt models and training)	+	Weak: very limited number of articles	(Turner et al. 2005; Rothman et al. 2007; Mwanahamuntu et al. 2011)	
**Institutional structures and infrastructure including financial resources and medical supplies**	Location, setting (this includes both accessibility and appropriateness)	±	Strong: range of settings and programmes	(Rothman et al. 2007; Dillard et al. 2010; Kumakech et al. 2015)	Careful consideration should be given to location(s), according to patient needs and circumstances
	funding to set up and sustain services	±	Moderate: range of settings and programmes but limited number of articles	(Mccarthy et al. 1992; Stringari-Murray et al. 2003; Hennessy et al. 2007; Jonsson et al. 2011; Moon et al. 2012)	
	Financing arrangements enabling access to (rather than being a barrier to) integrated services—according to country context e.g. insurance, free care	±	Moderate: range of programmes mainly but not exclusively US,	(Stringari-Murray et al. 2003; Bouis et al. 2007; Finkelstein et al. 2011)	
	Drug supply and availability; equipment	±	Moderate: several articles, range of settings	(Cheever et al. 2011; Finkelstein et al. 2011; Grenfell et al. 2012)	
**Leadership/stewardship/Procedures/ organizational culture**	Leadership, Lesson-learning and scale up, commitment and buy in from senior leaders, Buy in/acceptance of model and treatment from front line managers and staff, Resistance to change—presence or lack	±	Strong: range of settings and programmes	(Hoffman et al. 2004; Mwanahamuntu et al. 2011; UNAIDS, 2011; Moon et al. 2012)	An important precondition for implementing integration is the presence of high level commitment from the start, effective management structures and processes that are able to adapt and buy-in from front line users. Promoting change of organizational culture through dialogue, training, relationship building and appropriate use of knowledge and protocols will be important. Constant adaptation and lesson learning is essential to ensure that integration policy is fit for purpose.(this can be through monitoring and evaluation, reflection or other tools for systems (rather than programme) assessment)
	Structural and programme design facilitators and barriers: In/flexibility, availability, algorithms, checklists, Tools, guidelines and protocols including for referral and follow up; treatment regimen (simple vs complex);	±	Moderate: several articles, range of settings	(Kobayashi and Standridge 2000; Clanon et al. 2005; Rothman et al. 2007; Adeyemi et al. 2009; Goodroad et al. 2010; Adams et al. 2011; Curran et al. 2011; UNAIDS 2011; Odafe et al. 2013; Edwards et al. 2015; Kumakech et al. 2015)	
	Techniques and procedures/treatment (having or not having access to appropriate, timely, techniques)	±	Strong: range of programmes and settings	(Turner et al. 2005; Tran et al. 2012)	
	Different organizational culture (e.g. ‘behavioural vs medical’)	−	Weak: limited number of studies, mainly from USA in regards to mental health or substance misuse	(Cheever et al. 2011)	

‘+’,  facilitator, ‘−’  barrier.

The presence of a particular system feature may promote integration and its absence can obstruct it. The analysis demonstrated the importance of barriers and facilitators located outside the health system, including societal norms and patient preferences which critically affect how the systems operate; these are briefly presented in a subsequent section.

#### Collaborations and relationships

The first cross-cutting theme concerned collaboration and coordination as a facilitator or barrier to integration, including aspects particularly relevant to the service delivery, workforce and governance building blocks, but also to some extent the other blocks. Such linkages were important in all areas studied, including formal and informal relationships among providers and specialties (Feingold and Slammon 1993; Wright and Shuff 1995; Lemmon and Shuff 2001; Dodds *et al.* 2004; Bouis *et al.* 2007; Wood 2008; Moon *et al.* 2012), between patients and providers (Nebelkopf and Penagos 2005; Lucas *et al.*, 2007; Achmad *et al.* 2009; Egan *et al.* 2011) and with families and communities. Families and communities were especially important when integrating with services for substance use disorders and mental health issues (Feingold and Slammon 1993; Nebelkopf and Penagos 2005), which related primarily to their roles in awareness raising and peer education, and where community based organizations provided services (Wood and Austin, 2009; Mwanahamuntu *et al.* 2011; UNAIDS, 2011; Chamie *et al.* 2012; Khozaim *et al.* 2014).

Several aspects of this theme should be highlighted. First, there are multiple ways to achieve good communication and collaboration, varying according to context, including institutional processes, norms and culture (Wood 2008; Finkelstein *et al.* 2011). For example, co-location could facilitate communication (Dillard *et al.* 2010), but may be insufficient on its own (Lombard *et al.* 2009) and may sometimes even impede collaboration (Grenfell *et al.* 2012). Effective information sharing, using shared electronic records systems (Lombard *et al.* 2009; Inouye *et al.* 2011; Mwanahamuntu *et al.* 2011) and other information sharing agreements (Woods *et al.* 1998), facilitated communication, whereas restrictive regulations governing information sharing may be, or be perceived to be, a barrier (Lombard *et al.* 2009).

Second, mechanisms designed to improve communication may impose transaction costs, e.g. where there is an increased number of meetings between providers or different types of health worker (Clanon *et al.* 2005; Lombard *et al.*, 2009; Curran *et al.* 2011; Grenfell *et al.* 2012). Several studies report having a dedicated team member—who might or might not be a clinician, e.g. a nurse tasked with coordination. This person, who was recognizable to those involved, facilitated referrals to necessary services and information exchange between providers (Andersen *et al.* 2003), or other team members, and acted as ‘go to’ person. Thus, Tetrault *et al.* (2012), in an American study describing the integration of opioid dependency counselling and treatment with HIV care, highlighted the importance of having a nurse who acted as a ‘buprenorphine/naloxone coordinator’, ‘who became the “face” and the “glue” of the programme to patients, staff and providers’. Where this member of staff was additional to existing human resources, it addressed the time demands on existing staff imposed by additional interactions (Kobayashi and Standridge 2000). One study conducted in the USA found that the required level of coordination was difficult to maintain where the workload was low: when not routinely used, systems built on multiple interconnecting relationships among service providers tend to disintegrate, while having only one coordinator poses a risk if they leave (Lemmon and Shuff 2001).

Third, having a designated coordinator was useful not just for organizing meetings, shared services and facilitating communication between providers (Andersen *et al.* 2003). Designated coordinators, or a case management cadre (Bouis *et al.* 2007), were valuable where the complexity of care required a level of engagement and case management that clinicians were not able to give (Bouis *et al.* 2007; Egan *et al.* 2011). Case management was, however, mentioned as a facilitator predominantly in high-income settings, perhaps because adequately skilled staff were more easily identifiable in such settings or the resources were available to employ them. They also tended to feature in studies of integration of HIV with substance use or mental health disorders. These individuals could offer assistance beyond the health system, e.g. with housing (Schwartz *et al.* 1994).

Finally, strong relationships are built on mutual respect (Kobayashi and Standridge 2000) and trust (Lemmon and Shuff 2001; Krusi *et al.* 2009). These factors are especially important in strengthening relationships between staff and patients: many articles emphasized the importance of strong, respectful, trusting relationships in encouraging access and adherence, to services (Nebelkopf and Penagos 2005; Bouis *et al.* 2007; Achmad *et al.* 2009; Egan *et al.* 2011). These relationships are helped by taking into account cultural and language issues (Wood and Austin 2009). Similarly, ensuring adequate, appropriate [including culturally appropriate (Ibrahim *et al.* 2009) and non-judgemental (Altice *et al.* 2003)] patient-centred information and awareness was also found to be a facilitator.

#### Health workers, availability, roles and incentives

The availability and deployment of appropriately trained health workers was an important facilitator; in turn, the lack of appropriately trained workers created substantial barriers. These themes underpin all aspects of service delivery and health system governance.

The staff involved may already have the appropriate skills and expertise; e.g. psychiatrists who were knowledgeable about HIV and the interface between mental health, substance use and HIV (Bouis *et al.* 2007), or conversely, HIV clinics where there was already experience of offering multiple services and dealing with psychosocial issues (Egan *et al.* 2011). Multi-professional teams were mentioned as a facilitator (Wood 2008; Edwards *et al.* 2015; Kumakech *et al.* 2015), primarily in high-income settings. Studies from low-income countries (LICs) often identified a lack of appropriately trained staff, such as a shortage of pathologists (Khozaim *et al.* 2014), staff able to perform pap smears (Rosa-Cunha *et al.* 2011) or medical staff in substance use disorder clinics who were sufficiently knowledgeable about HIV (Rothman *et al.* 2007). Staff turnover was also problematic (Cheever *et al.* 2011; Nyabera *et al.* 2011).

Task-shifting was recognized as a way to overcome human resource constraints, particularly in low-income settings, e.g. using nurses for cervical cancer screening in Africa (Moon *et al.* 2012; Ramogola-Masire *et al.* 2012; Odafe *et al.* 2013; Khozaim *et al.*, 2014; Martin *et al.* 2014; Anderson *et al.* 2015), but also in one article in a high-income (USA) setting. Adams *et al* (2012) reported improved outcomes with task shifting in nurse-led depression management. The availability of staff for support functions including laboratories, equipment maintenance and logistics, were also cited as facilitators, or barriers (where lacking) (Chamie *et al.* 2012; Conners *et al.* 2012; Moon *et al.* 2012; Huchko *et al.* 2011; Khozaim *et al.* 2014).

Where staff were available, but existing skills were not sufficient, training was commonly provided (Altice *et al.* 2004; Ibrahim *et al.* 2009; Huchko *et al.* 2011; Kieran *et al.* 2011; Nyabera *et al.* 2011; Horo *et al.* 2012; Moon *et al.* 2012; Ramogola-Masire *et al.* 2012; Hasin *et al.* 2013; Odafe *et al.* 2013; Khozaim *et al.* 2014; Martin *et al.* 2014; Anderson *et al.* 2015). Several articles reported staff training to manage co-morbidities effectively as a facilitator (Clanon *et al.* 2005; Huchko *et al.* 2011; Odafe *et al.* 2013; Khozaim *et al.* 2014; Kotwani *et al.* 2014; Martin *et al.* 2014; Anderson *et al.* 2015), and existing staff can be trained in specific techniques such as cervical cancer screening (Huchko *et al.* 2011; Horo *et al.* 2012; Ramogola-Masire *et al.* 2012; Martin *et al.* 2014; Anderson *et al.* 2015) with follow up supervision and training (Huchko *et al.* 2011; Horo *et al.* 2012; Martin *et al.* 2014). There were different training modalities: e.g. using specialist staff as mentors, [e.g. linking experienced buprenorphine/naloxone prescribers with less experienced ones (Finkelstein *et al.* 2011)], train-the-trainer programmes (Hasin *et al.* 2013) or peer-learning. Ongoing supervision, support or training by specialists—whether by joint meetings, case conferences (Clanon *et al.* 2005; Nebelkopf and Penagos 2005) or through telephone support was also identified by many articles as a facilitator. Insufficient staff training (Finkelstein *et al.* 2011) and lack of regular supervision (Martin *et al.* 2014) were cited as barriers. Training, however provided, inevitably carried resource and logistical implications so the lack of corresponding resources was also identified as a barrier (Tobias *et al.* 2005).

Technical skills and expertise were not the only important factors pertaining to staff. They were at risk of stress from the increased workload posed by integration policies, which created competing demands (Stopka *et al.* 2007). Staff fears and concerns, e.g. about being responsible for additional tasks (Curran *et al.* 2011), and communicating bad news to patients (Henry 2010; Conners *et al.* 2012), or their ability to perform new tasks, were mentioned as barriers to integration, as was the stigma that some care providers attached to certain marginalized groups—such as people living with HIV, drug dependence or mental health issues (Sanchez *et al.* 2012). In turn, the presence of motivated, interested and engaged staff facilitated delivery (Aharonovich *et al.* 2006; Proeschold-Bell *et al.* 2010; Nyabera *et al.* 2011). Again, training was important, in terms of staff awareness, to encourage cultural understanding or respect for other professional groups and disciplines (Kobayashi and Standridge 2000). Financial incentives were reported, in some studies, to facilitate staff retention (Mwanahamuntu *et al.* 2011), participation in training (Turner *et al.* 2005) or participation in an integrated programme (Rothman *et al.* 2007).

#### Institutional structures and resources to support integration

Another key theme that emerged to underpin integration could broadly be described as the requirement for a health system to have the right ‘hardware’: the right physical structures, commodities and funding, which were identified as facilitators or barriers that particularly related to the service delivery block but also workforce, and medicines and technology.

The existing infrastructure: location and setting of the services was by far the most significant characteristic within this theme. Location was important both in terms of accessibility and appropriateness. Co-location can facilitate integration by improving accessibility; e.g. Rothman *et al.* (2007) found that on-site primary medical care services were readily and frequently used by patients at a methadone programme. Kumakech *et al.* (2015) reported the benefit of integration to patients/clients, not only from convenience and saving time by only having to attend one appointment, but also collateral benefits of opportunistic screening and identification of diseases, and Dillard *et al.* (2010) noted the benefits in terms of scheduling and reducing transport time. Several studies confirmed the intuitive finding that accessibility was improved by setting up clinics close to where the main target population lives, or by using or adding outreach services to bring the services to the client or patient (Rothman *et al.* 2007; Dillard *et al.* 2010; Kumakech *et al.* 2015). However, co-location is not always universally appropriate or achievable. Patients may find co-location reassuring, such that they could safely disclose both substance use disorders and HIV diagnoses (Dillard *et al.* 2010; Egan *et al.* 2011), but if not managed sensitively and appropriately, co-location can be a barrier e.g. in terms of confidentiality (Kobayashi and Standridge 2000; Cooperman *et al.* 2007; Lombard *et al.* 2009) or where some groups are stigmatized (Sanchez *et al.* 2012).

Space and facilities also need to be fit-for-purpose (Lombard *et al.* 2009; Nyabera *et al.* 2011) and appropriate, e.g. to enable longer, confidential counselling sessions to patients or to administer supervised ART (Lucas *et al.* 2007; Lombard *et al.* 2009; Nyabera *et al.* 2011; Khozaim *et al.* 2014). Offering medication in an ‘office’ rather than a clinic environment can be attractive to patients/clients in offering non-medicalized space, promoting autonomy and being separate from other services (Clanon *et al.* 2005; Egan *et al.* 2011).

Limited access to drugs, perhaps because of different licensing requirements (Cheever *et al.* 2011; Finkelstein *et al.* 2011) and supply problems (Grenfell *et al.* 2012) could be barriers, as could the lack of other materials and properly maintained equipment, especially in resource-constrained settings (Nyabera *et al.* 2011; Hasin *et al.* 2013; Odafe *et al.* 2013; Khozaim *et al.* 2014).

Not surprisingly, given these considerations, and those pertaining to the workforce, a small but significant number of articles referred to issues relating to funding to set up and sustain integrated services (with availability of funding acting as a facilitator (Stringari-Murray *et al.* 2003; Jonsson *et al.* 2011; Moon *et al.* 2012) while its absence was a barrier (McCarthy *et al.* 1992; Hennessy *et al.* 2007).

#### Leadership, stewardship, management and organizational culture

The fourth major cross-cutting theme concerned leadership, stewardship and culture and highlights the central role of these health systems characteristics in facilitating or inhibiting integration. The theme has three dimensions. First, key factors that are seen to facilitate integration relate to leadership, including political will to implement integration (Inouye *et al.* 2011), defining integration as an explicit goal and having a vision and clearly defined strategy for implementing it. This is demonstrated by having commitment and buy-in from high-level policy makers, reported at US state level (Hoffman *et al.* 2004), and nationally in LICs (Mwanahamuntu *et al.* 2011; UNAIDS 2011; Moon *et al.* 2012).

Second, integration is facilitated where these strategies are enabled by structural and programme design features. This includes support for integrated models by senior managers at operational level (Rothman *et al.* 2007; Curran *et al.* 2011), in leading facilities. Strong leadership can ensure that this vision is shared among multiple stakeholders (Dodds *et al.* 2004; Odafe *et al.* 2013), valuing successful locally-led initiatives (UNAIDS 2011). This is particularly important during scale up, where the viability and sustainability of particular models positively influences their diffusion (Woods *et al.* 1998; Goodroad *et al.* 2010) and favours lesson-learning (Inouye *et al.* 2011); Buy-in by frontline managers and staff is considered a critical facilitator.

Integration of the management of different chronic conditions was facilitated by formulating explicit guidelines, protocols, checklists and algorithms (Kobayashi and Standridge 2000; Clanon *et al.* 2005; Adeyemi *et al.* 2009; Goodroad *et al.* 2010; Adams *et al.* 2011; UNAIDS 2011; Odafe *et al.* 2013; Edwards *et al.* 2015; Kumakech *et al.* 2015) or inhibited by the absence of guidelines (Grenfell *et al.* 2012; Edwards *et al.* 2015). Differences in administrative processes (e.g. for prescribing, data recording and sharing) among providers was identified as a barrier in many studies, undermining integration and making it difficult to evaluate progress.

Third, high-level support for integration is insufficient without a ‘change in organizational culture’. This requires the creation of a shared vision and proactive engagement by different actors, including users and their families. This is often less tangible and is most obvious when it is absent, e.g. where there is a clash of organizational cultures, as in cases where one service is based on a behavioural, patient-centred approach while another has a more medicalized model. Thus, although the introduction of Directly Administered Antiretroviral Therapy at one methadone clinic was associated with improved dialogue about HIV and better collaboration (Lucas *et al.* 2004), Cheever *et al.* (2011) noted the challenges posed by competing cultures of those involved in the management of HIV and substance use disorders.

#### The patient, families and the community at the centre of integration

Our analysis highlighted a number of thematic barriers and facilitators that related to patients, personal and social context and norms, their health-seeking behaviour and the health systems responses to their needs. Stigma was especially important as integration often involved extending services to marginalized groups (Kobayashi and Standridge 2000; Altice *et al.* 2003; Curran *et al.* 2011; UNAIDS, 2011; Mwanahamuntu *et al.* 2011, 2013; Chamie *et al.* 2012), in some case double stigma for attending both mental health and HIV clinics (Curran *et al.* 2011). Many examples related to the provision of care for HIV for people engaged in substance use. Fear among clients was a commonly cited barrier to use of integrated services: fear of dual diagnoses (Mwanahamuntu *et al.* 2011; Ibrahim *et al.* 2009; Kumakech *et al.* 2015), side effects of treatment (Egan *et al.* 2011 ; Rosa-Cunha *et al.* 2011; Grenfell *et al.* 2012) or breach of confidentiality (Proeschold-Bell *et al.* 2010). Recognizing the challenges that the health system faces in tackling these barriers, some studies (Lucas *et al.*, 2004; Clanon *et al.* 2005; Bouis *et al.* 2007; Egan *et al.* 2011; Monroe *et al.* 2012) from the USA identified patient peer-to-peer support as facilitating use of integrated care services. Other studies, particularly conducted in low resource settings, highlighted the importance of including families and partners (Cartter *et al.* 1990; Feingold and Slammon 1993; Dodds *et al.* 2004; Edwards *et al.* 2015; Kumakech *et al.* 2015) in educational and awareness programmes.

## Discussion

In recent years, integration of services conventionally provided separately has attracted increasing attention. This review synthesizes evidence on health system (exogenous) factors that facilitate or obstruct integration of services for those with HIV and NCDs. These experiences indicate that achieving successful integration depends, to some extent, on the presence or absence of a range of health system-related factors that can facilitate or obstruct progress. These are often not made explicit when planning how to integrate programmes.

Five cross-cutting themes closely related to health systems design and operation have been shown to facilitate or impede integration of health services for those suffering from HIV and from other chronic conditions. These are: (1) formal and informal communication, relationships and collaboration; (2) availability of trained and incentivized health workers, with appropriate roles, (3) institutional structures and resources supportive of integration; (4) effective leadership and management and a supportive organizational culture; and (5) a patient-centred health system.

Communication, building relationships and collaboration emerge as especially important. They involve a multitude of formal and informal relationships, vertical and horizontal links within teams and between teams, across different levels of care, e.g. coordinated management and clear referrals. Relationships are enhanced in situations where there is trust, with the mutual exchange of information required to manage complex cases, especially where guidelines (mostly designed for ‘typical’ cases) are less useful. One interpretation is that where mutually beneficial relationships exist, any issues that obstruct integration can be negotiated and overcome in a flexible manner.

The practical steps involved in creating supportive models of communication and collaboration vary, affected by context such as institutional processes, norms and culture. However, one important message was that integration imposed transaction costs; communication and collaboration are not cost free. Consequently, there are benefits in having a dedicated team member, known to all those involved as the ‘face’ of the programme, or a ‘go-to’ when problems arise. This ensures a consistent buy-in in relation to integration. A further characteristic identified as facilitative in several studies was engagement with the wider social and other networks on which patients depend.

The availability and deployment of appropriately trained health workers greatly facilitated integration: where this was not achieved, it created important barriers. The development of expertise by those specialized in one area, such as HIV, in the management of manifestations of other chronic conditions was helpful in some settings. Mobilizing and incentivizing staff working within the system to form part of an integrated treatment model or training in new skills while ensuring mentoring and supervision facilitated integration. However, returning to the interdependence of health systems building blocks, major barriers existed where other elements of the health system needed to support frontline providers were missing. For example, many complex activities, such as cancer screening, depend on the availability of well-equipped laboratories and trained laboratory staff. Some benefits could be achieved by task-shifting, especially in systems that have traditionally been medically dominated, with nurses taking on extended roles (although in this respect only moving towards what is now the norm in many high-income settings). In this respect, they were adopting practices already well established in some high-income countries where extended roles of nurses and other non-medical professionals are much better established (McKee *et al.* 2006).

Physical and institutional structures to support integration emerged as important. Accessibility and co-location of facilities appeared advantageous, especially for patients who needed to make fewer journeys but also because it increased scope for opportunistic interventions. In some cases this helps to avoid stigmatization, as might arise when patients were seen attending separate HIV services, but this was not always the case. Thus, outreach services were better at reaching people suffering multiple disadvantages. It was also important to have appropriate infrastructure, such as rooms that offered opportunities for counselling and near patient testing equipment, and facilities designed to welcome patients.

Leadership and organizational culture emerged as essential underlying characteristics often critically influencing the extent to which other inputs (collaborative proactive, trained personnel, appropriately used resources) work well within integrated services. This reflects growing recognition of the importance of effective governance in design and implementation of health systems interventions (Balabanova *et al.* 2013; Kuruvilla *et al.* 2014).

The presence of explicit rules guiding practice (e.g. guidelines on prescribing and referrals, care checklists), consistent across facilities and organizations, coupled with mechanisms for exchange of information and lesson-learning also emerged as important facilitative set of factors. This involves fostering an organizational culture compatible with holistic models of care and accepting the need for reconfiguration of existing structures and routines, which may be disruptive for staff and managers in the short term.

Although the focus of the review was on health system-related factors affecting integration, the results also highlight the importance of patient preferences, family support, social norms and culture (Isaakidis *et al.* 2013) in integration of services. This is a reminder of how the patient has agency in shaping the treatment interaction, reflecting the concept of people-centred systems (Sheikh *et al.* 2014) This process (Sheikh *et al.* 2014) is in turn dependent on the context in which it is embedded. Some of these factors can be addressed through health systems interventions—improving communication, adapting services and involving patients—while others, such as stigma towards particular diseases (or rather people who have them), may require long term measures to change attitudes and culture. Importantly, while culture and preferences are noted in many of the studies reviewed, especially in the context of adherence to formally prescribed treatment, discussion of other values that shape health care delivery such as the right to health, dignity in care and equity is less common in how they impact on effective integration and health outcomes.

To the best of our knowledge, this is the first study to look systematically at health system barriers and facilitators to integration of services for HIV/AIDS and those for such a wide range of other conditions. Haregu *et al.* (2015) e.g. concluded that whilst the epidemiological, clinical and management related evidence for the integration of HIV/AIDS and NCDs is strong, and the data available suggested, the integrated approach was feasible, effective, efficient and acceptable, the evidence base to inform decisions related to integration of HIV with NCDs was still limited. Moreover, the overarching messages are consistent with other studies that have looked at integration with specific conditions. The importance of patient involvement, e.g. emerged in a review of integrating HIV services with mental health issues (Kaaya *et al.* 2013) as did the importance of adequate staff capacity. A review of approaches to integrating TB and HIV services also identified the need for adequate trained staff and appropriate infrastructure (Legido-Quigley *et al.* 2013).

## Limitations

Our review was complicated by the existence of considerable diversity in the terminology employed in the literature on health service integration. Thus, ‘integrated care’ is often used to describe services that are merely ‘co-located’, and there is no clarity about how to define different degrees of integration (partial or full). The literature was also limited by the scarcity of studies that looked at outcomes of integration, such as improved health or greater patient responsiveness and satisfaction. In practice, most studies focused on process measures. It was clear that most of the models evaluated were contextually bounded, limiting the generalizability of any findings (Gilson 2012). The majority of evaluative studies were also assessed as having a high risk of bias. Few studies explicitly considered those aspects of the context that would influence wider applicability. Moreover, our focus on the health system could have lead us to miss important factors influencing integrated programmes, such as community dynamics and societal barriers to care faced by particular patient groups. Given that the majority of studies included in this review were conducted in the high-income settings, especially the USA, the findings may not be generalizable to low and middle-income settings. However, the overarching themes found support in by examples from all types of settings and country contexts. This suggests that, with caution, the review can generate lessons relevant to countries of all income levels. Although the specific facilitators and barriers will vary across settings, and further research is clearly needed in LMICs, the underlying institutional structures, processes and culture required to support integration, are likely to reside in the same health system domains.

We began by using a commonly used health system framework (the WHO building blocks) to guide the first step of data extraction and to organize the data, reducing the variability between reviewers. However, we found that the framework was of limited value in interpreting the findings and may lead to oversimplification of complex processes and institutional behaviour. In practice, many of the key issues cut across several of the building blocks, as would indeed be expected from a systems perspective. This was particularly problematic in the case of governance which is often seen as the overarching health system function (WHO 2007). Thus in our analysis, governance subthemes—e.g. effective regulatory and management frameworks, coordination and relationship and knowledge generation and sharing—were identified across all building blocks. Thus, identifying system-wide patterns of institutional structures, processes and relationships may represent better the review findings.

### Implications for policy

This review offers a reminder that integration is not a quick fix. Importantly, failure to achieve integration may be less to do with ineffective programmes *per se* but rather with insufficiently supportive health systems within which these interventions are located. Thus, integration requires investment beyond particular programmes, building supportive systems in areas of collaborative practice and professional training, while recognizing the need for holistic care for co-morbidities, and appropriate health system ‘hardware’. Integration, therefore, requires effective governance, political will and skilled management and not simply allocating finances differently. Effective collaboration, teamwork, creating networks and engaging a wide range of stakeholders to support policy implementation is now seen as a hallmark of a well-functioning health system.(WHO 2007) This review suggests that building relationships and trust, increasingly seen as essential in health systems (Gilson 2003), are important underlying drivers of effective coordination and collaboration, and are also fundamental for service delivery integration. However, in reality, achieving trust often remains more rhetorical than pursued in practice, with the implementation of integration therefore failing to realize the expected benefits. Consequently, an appropriate culture is required to accommodate new models of care, build relationships between specialities and the acceptance of holistic care paradigms by patients and providers.

Our review found that most supportive factors involved several health system building blocks. Thus, investment will often be needed in several areas, ensuring that it is aligned with the common objective and there is attention to realizing synergies.

Although there is no universal blueprint for achieving integration in all settings, some practical solutions emerge. The role of service coordinators is key and can be strengthened. Standardized guidelines or checklists can be useful in all income settings but especially where resources are limited, helping to overcome variations in access and quality. Conversely, providing a more individually tailored health services targeted to specific contexts, settings and populations, also offers promise for improving access to and quality of care. The expectations need to be managed carefully. Many of the changes of practice and adaptation of infrastructure even if efficient in the long run, involve significant start-up costs.

These findings emphasize the need to consider carefully how to support patients in the care process, respond to their needs and preferences and build relationships with all relevant stakeholders, when implementing integrated delivery models. If integration is applied too rigidly, it may exclude or stigmatize particular patients whose needs do not fit the integrated models. A key point is that these models should build on and enhance informal relationships and social interactions. Increasing awareness and reducing stigma, especially that associated with HIV and substance use amongst the wider population, may influence substantially the extent to which integration will be feasible and lead to better health outcomes.

### Implications for research

This review confirmed the need for research not only on endogenous factors affecting service delivery integration, but also on factors located outside the specific programme. This would require studies of the complex interrelationships and processes with which the health system is involved. It is also important to understand the specific challenges facing poor and marginalized people who can benefit from integrated programmes, exploring holistic approaches that respond to these needs.

## Funding

Funding was received from The Joint United Nations Programme on HIV/AIDS (UNAIDS) grant number ADDEVH48, which covered for HL-Q’s salary and payment for accessing papers.

*Conflict of interest statement*. None declared.

Box 1 Domains of integration

Drawing on (Groene and Garcia-Barbero 2001; Briggs and Garner 2006; WHO 2008a,b; Atun et al. 2010a,b; Shigayeva and Coker 2015)

- Integration across disease programmes (clinically related diseases)
- Integration across disease programmes (clinically different diseases), e.g.:
    □ Integration across high burden conditions (e.g. HIV, malaria, TB) to reduce impact of co-infections
- Integration between vertical (disease-specific) and horizontal (system-wide) programmes, which may involve:
    □ Integration of interventions within a ‘building block’ of the health system (e.g. integrated staff training, financial and organizational management etc.)
    □ Integration across one or more building blocks of the health system (e.g. human resource policies and governance initiatives)
    □ Integration across ‘service functions’: of inputs, of different levels of service delivery, of management and operational decisions and technology
- Integration across public health programmes and health service interventions, e.g.:
    □ integration between MNCH, family planning, through trained community health workers, and health promotion.
- Integration across activities in the health systems and other sectors (e.g. treatment combined with educational interventions and community mobilization)

## Supplementary Material

Supplementary Table

Supplementary Material
