# Supplementary material for: Health systems facilitators and barriers to the integration of HIV and chronic disease services: a systematic review
Source: Health Policy Plan. 2017 Nov 24;32(Suppl 4):iv13–26. doi: 10.1093/heapol/czw149 (PMC5886067; doi:10.1093/heapol/czw149)
Supplement: Supplementary Material [file czw149_supplementary_material_12_august_2016.docx]

# Supplementary material

**Table A1. List of chronic diseases, long term conditions, and related services included in the review**

| Alzheimer Disease |
| --- |
| Arthritis (Osteoarthritis, Rheumatoid Arthritis, Sacroiliitis, Spondylarthritis, Spondylarthropathies) |
| Asthma |
| Breast cancer |
| Cervical cancer |
| Colorectal cancer |
| Coronary Heart Diseases |
| Chronic Cardiovascular Diseases |
| Cerebrovascular Diseases (stroke) |
| Chronic Obstructive Pulmonary Disease |
| Depressive Disorders |
| Diabetes Mellitus Type 2 |
| Hepatitis C services |
| Hypertension |
| Lung cancer |
| Mental health services or alcohol and substance use disorders |
| Prostatic cancer |
| Vascular Dementia |

**Table A2 – Overview of included studies**

There were 153 studies presented in the 150 papers included.

| **Regions** | **% (n)** |
| --- | --- |
| N America (US and Canada) | 58.8 (90) |
| Africa | 25.5 (39) |
| Europe | 9.2 (14) |
| Asia | 3.3 (5) |
| Australia | 0.7 (1) |
| S America | 2.0 (3) |
| Africa and S.America | 0.7 (1) |
| **Number of studies integrating HIV with one or more additional defined chronic conditions^$^** | **%(n)** |
| 1 chronic condition | 71.9 (110) |
| 2 chronic conditions | 19.6 (30) |
| 3+ chronic conditions | 8.5 (13) |
| **Study design** | **%(n)** |
| Descriptive | 28.1 (43) |
| Cross-sectional | 15.0 (23) |
| Cohort | 14.4 (22) |
| Retrospective review | 12.4 (19) |
| RCT | 8.5 (13) |
| Qualitative | 7.8 (12) |
| Intervention study | 3.9 (6) |
| Mixed Methods | 3.3 (5) |
| Case Series | 2.6 (4) |
| Case-Control | 1.3 (2) |
| Cost analysis | 1.3 (2) |
| Rapid assessment | 0.7(1) |
| Other | 0.7 (1) |
| **Number of participants covered** | |
| Median (min – max) | 133 (2 – 58,000) |
| **Proportion of integrated chronic conditions covered ^ % (n)** | |
| Substance or alcohol misuse | 47.7 (73) |
| Mental Health | 29.5 (45) |
| Cancer | 19.0 (29) |
| Hepatitis or liver disease | 10.4 (11) |
| Diabetes, hypertension or CVD | 11.1 (17) |
| Chronic kidney disease | 0.7 (1) |

*$besides the HIV support delivered*

*^several integration models included more than one chronic condition*

**Table A3: Adapted data extraction table (attached as a separate Excel file)**

**Table A4 : Key emerging facilitators and barriers for each building block of health system (not exhaustive)**

| Block | Facilitators | Barriers |
| --- | --- | --- |
| Service delivery | N^[[1]](#footnote-1)^=99 (63.9%) | N=86 (55.5%) |
|  | collaboration between providers | Referral and linkage to follow up treatment |
|  | Coordination (of services) and case management | Bureaucracy, lack of access to data limits effectiveness, follow up and evaluation |
|  | Workforce -training and supervision of staff | Staff education, skills and knowledge |
|  | Accessibility – location, setting | Resources – set up and sustaining and expanding services, time |
|  | Techniques/treatment - quick responsive support/treatment, testing, results |  |
| Health workforce | N=76 (49%) | N=40 (25.8%) |
|  | Staff training, education, skills | - Staff education, skills and experience |
|  | Availability of staff including specialist staff | - Availability of staff |
|  | Necessary/facilitative staff culture, interest, enthusiasm | Lack of staff interest, understanding or acceptance of behaviour changes needed |
|  | Collaboration/communication between services | Poor collaboration between providers |
|  | Coordination/navigation (including by a coordinator) |  |
|  | Multidisciplinary teams |  |
| Information | N=40 (25.8%) | N=22 (14.2%) |
|  | Adequate (and adequate quality) data collection/recording | Insufficient/poor data collection |
|  | Appropriate, accessible databases/electronic record systems. | Difficulties in accessing data due to different provider systems |
|  | Mobile phones for telehealth, patient follow up, staff training | Lack of written guidelines and shared protocols |
| Medical products, vaccines & technologies | N=36 (23.2%) | N=35 (22.6%) |
|  | Effective medication, minimising adverse effects | - Drug interactions and side effects |
|  | - Sustainable, uninterrupted drug supply | - Drug availability and supply |
|  | Simple treatment regimen | - Complex treatment regimens |
|  | - Access to equipment and technology (including maintenance of equipment) | Lack of appropriate equipment/ongoing maintenance and repair |
| Financing | - N=34(21.9%) | - N=24 (15.5%) |
|  | Funding . e.g. to pay for set up and sustaining services | Costs for set up, sustaining and expanding (e.g., because more people identified) |
|  | Insurance coverage and reimbursement to enable client access (including for non-insured) | Insurance coverage |
|  | Free care including medicines |  |
|  | Financial incentives for providers to take part |  |
| **Leadership/governance** | N=32 | N=8 |
|  | Communication, Coordination/ coordinator, Effective collaboration | Communication |
|  | Structural & programme design facilitators | Structural & programme design barriers, Inflexibility, Lack of policies and standards, perceived as increasing workload |
|  | Leadership, Lesson-learning and scale-up, Commitment and buy in from senior leaders, Buy in / acceptance of model and treatment from front line managers and staff, Local champions | Lack of leadership and commitment, Resistance to change /  Different organisational culture (e.g. “behavioural vs medical”) |
|  | Strong relationships between providers and stakeholders (trust, respect, informal links) |  |

**Box A1. Risk of bias**

For randomized studies the Cochrane risk of bias tool was used. This tool includes selection bias, study design, confounders, blinding, data collection methods and withdrawals and drop-outs. For observational study designs, risk of bias will be assessed using a simple proforma for three domains: selection bias, information bias (differential misclassification & non-differential misclassification) and confounding. Risk of bias for each domain was assessed as either low, unclear or high. Studies which had a low risk of bias in all domains were classified as having a low overall risk of bias.

Qualitative studies were evaluated using an adapted version of a checklist used in a previous series of mixed methods systematic reviews incorporating both quantitative and qualitative studies [^23^](file:///H:\My%20Documents\My%20papers%20&amp;%20conferences\Submitted%20papers\Integration%20HIV%20and%20NCD\Paper\Revision%20June-Aug%202016\Supplementary%20file%202016.08.11.docx#_ENREF_23)^,^[^24^](file:///H:\My%20Documents\My%20papers%20&amp;%20conferences\Submitted%20papers\Integration%20HIV%20and%20NCD\Paper\Revision%20June-Aug%202016\Supplementary%20file%202016.08.11.docx#_ENREF_24) and that was first presented in a systematic review conducted by some of the author of a previous review. The risk of bias was assessed using a range of domains assessing background, aims and objectives of the study, context, sampling, description of data collection and analysis, reliability, clarity about how conclusions were derived, reflexivity, generalizability and ethics. Risk of bias for each domain was assessed as either low, medium or high. Studies which had a low risk of bias in all domains were classified as having a low overall risk of bias. Due to the substantial heterogeneity in study design, exposures, participants and outcomes between included studies, a meta-analysis was not carried out.

**Research Protocol:**

**Integrating Non-Communicable Diseases and HIV services: a systematic review**

Helena Legido-Quigley, Nicola Watt, Louise Sigfrid, Sue Hogarth, Will Maimaris, Pablo Perel, Dina Balabanova, Kent Buse, Martin McKee, Peter Piot

London School of Hygiene and Tropical Medicine and UNAIDS

Contents

[Introduction 2](file:///H:\My%20Documents\My%20papers%20&amp;%20conferences\Submitted%20papers\Integration%20HIV%20and%20NCD\Paper\Revision%20June-Aug%202016\Supplementary%20file%202016.08.11.docx#_Toc350795262)

[Objectives 3](file:///H:\My%20Documents\My%20papers%20&amp;%20conferences\Submitted%20papers\Integration%20HIV%20and%20NCD\Paper\Revision%20June-Aug%202016\Supplementary%20file%202016.08.11.docx#_Toc350795263)

[Objective 1 Models of integration 3](file:///H:\My%20Documents\My%20papers%20&amp;%20conferences\Submitted%20papers\Integration%20HIV%20and%20NCD\Paper\Revision%20June-Aug%202016\Supplementary%20file%202016.08.11.docx#_Toc350795264)

[Objective 2 Health System Barriers and Facilitators 4](file:///H:\My%20Documents\My%20papers%20&amp;%20conferences\Submitted%20papers\Integration%20HIV%20and%20NCD\Paper\Revision%20June-Aug%202016\Supplementary%20file%202016.08.11.docx#_Toc350795265)

[Conceptual framework 4](file:///H:\My%20Documents\My%20papers%20&amp;%20conferences\Submitted%20papers\Integration%20HIV%20and%20NCD\Paper\Revision%20June-Aug%202016\Supplementary%20file%202016.08.11.docx#_Toc350795266)

[Definition of Integration 4](file:///H:\My%20Documents\My%20papers%20&amp;%20conferences\Submitted%20papers\Integration%20HIV%20and%20NCD\Paper\Revision%20June-Aug%202016\Supplementary%20file%202016.08.11.docx#_Toc350795267)

[Health Systems Conceptual Framework 4](file:///H:\My%20Documents\My%20papers%20&amp;%20conferences\Submitted%20papers\Integration%20HIV%20and%20NCD\Paper\Revision%20June-Aug%202016\Supplementary%20file%202016.08.11.docx#_Toc350795268)

[Measures of effectiveness of integration 5](file:///H:\My%20Documents\My%20papers%20&amp;%20conferences\Submitted%20papers\Integration%20HIV%20and%20NCD\Paper\Revision%20June-Aug%202016\Supplementary%20file%202016.08.11.docx#_Toc350795269)

[Methods 6](file:///H:\My%20Documents\My%20papers%20&amp;%20conferences\Submitted%20papers\Integration%20HIV%20and%20NCD\Paper\Revision%20June-Aug%202016\Supplementary%20file%202016.08.11.docx#_Toc350795270)

[Type of chronic conditions 6](file:///H:\My%20Documents\My%20papers%20&amp;%20conferences\Submitted%20papers\Integration%20HIV%20and%20NCD\Paper\Revision%20June-Aug%202016\Supplementary%20file%202016.08.11.docx#_Toc350795271)

[Search strategy 7](file:///H:\My%20Documents\My%20papers%20&amp;%20conferences\Submitted%20papers\Integration%20HIV%20and%20NCD\Paper\Revision%20June-Aug%202016\Supplementary%20file%202016.08.11.docx#_Toc350795272)

[Search and retrieval of studies 7](file:///H:\My%20Documents\My%20papers%20&amp;%20conferences\Submitted%20papers\Integration%20HIV%20and%20NCD\Paper\Revision%20June-Aug%202016\Supplementary%20file%202016.08.11.docx#_Toc350795273)

[Data extraction 7](file:///H:\My%20Documents\My%20papers%20&amp;%20conferences\Submitted%20papers\Integration%20HIV%20and%20NCD\Paper\Revision%20June-Aug%202016\Supplementary%20file%202016.08.11.docx#_Toc350795274)

[Quality appraisal of quantitative studies 8](file:///H:\My%20Documents\My%20papers%20&amp;%20conferences\Submitted%20papers\Integration%20HIV%20and%20NCD\Paper\Revision%20June-Aug%202016\Supplementary%20file%202016.08.11.docx#_Toc350795275)

[Quality appraisal of qualitative studies 8](file:///H:\My%20Documents\My%20papers%20&amp;%20conferences\Submitted%20papers\Integration%20HIV%20and%20NCD\Paper\Revision%20June-Aug%202016\Supplementary%20file%202016.08.11.docx#_Toc350795276)

[Presentation and synthesis of results 8](file:///H:\My%20Documents\My%20papers%20&amp;%20conferences\Submitted%20papers\Integration%20HIV%20and%20NCD\Paper\Revision%20June-Aug%202016\Supplementary%20file%202016.08.11.docx#_Toc350795277)

[References 9](file:///H:\My%20Documents\My%20papers%20&amp;%20conferences\Submitted%20papers\Integration%20HIV%20and%20NCD\Paper\Revision%20June-Aug%202016\Supplementary%20file%202016.08.11.docx#_Toc350795278)

[Appendix 1: Quality appraisal of evidence from quantitative studies using GRADE 10](file:///H:\My%20Documents\My%20papers%20&amp;%20conferences\Submitted%20papers\Integration%20HIV%20and%20NCD\Paper\Revision%20June-Aug%202016\Supplementary%20file%202016.08.11.docx#_Toc350795279)

[Appendix 2: Tool for quality appraisal of qualitative studies 12](file:///H:\My%20Documents\My%20papers%20&amp;%20conferences\Submitted%20papers\Integration%20HIV%20and%20NCD\Paper\Revision%20June-Aug%202016\Supplementary%20file%202016.08.11.docx#_Toc350795280)

[Appendix 3: Search strategy 13](file:///H:\My%20Documents\My%20papers%20&amp;%20conferences\Submitted%20papers\Integration%20HIV%20and%20NCD\Paper\Revision%20June-Aug%202016\Supplementary%20file%202016.08.11.docx#_Toc350795281)

[Integration Search Strategy for Medline 13](file:///H:\My%20Documents\My%20papers%20&amp;%20conferences\Submitted%20papers\Integration%20HIV%20and%20NCD\Paper\Revision%20June-Aug%202016\Supplementary%20file%202016.08.11.docx#_Toc350795282)

[Health Systems Search Strategy for Medline 13](file:///H:\My%20Documents\My%20papers%20&amp;%20conferences\Submitted%20papers\Integration%20HIV%20and%20NCD\Paper\Revision%20June-Aug%202016\Supplementary%20file%202016.08.11.docx#_Toc350795283)

# Introduction

Non-communicable Diseases (NCDs) together with HIV infection are among the major public health concerns worldwide. According to WHO, in 2008, an estimated 36 million of the 57 million global deaths (more than 60%) were due to noncommunicable diseases, principally cardiovascular diseases, cancers, chronic respiratory diseases and diabetes, including about 9 million deaths before the age of 60 [[1](file:///H:\My%20Documents\My%20papers%20&amp;%20conferences\Submitted%20papers\Integration%20HIV%20and%20NCD\Paper\Revision%20June-Aug%202016\Supplementary%20file%202016.08.11.docx#_ENREF_1)]. Nearly 80 per cent of those deaths occurred in developing countries. In African nations deaths from NCDs are projected to exceed the combined deaths of communicable and nutritional diseases and maternal and perinatal deaths as the most common causes of death by 2030 [[1](file:///H:\My%20Documents\My%20papers%20&amp;%20conferences\Submitted%20papers\Integration%20HIV%20and%20NCD\Paper\Revision%20June-Aug%202016\Supplementary%20file%202016.08.11.docx#_ENREF_1)].

In 2008, 33.4 million people were estimated to be living with HIV, 2.7 million to have acquired new HIV infection and 2.0 million to have died [[2](file:///H:\My%20Documents\My%20papers%20&amp;%20conferences\Submitted%20papers\Integration%20HIV%20and%20NCD\Paper\Revision%20June-Aug%202016\Supplementary%20file%202016.08.11.docx#_ENREF_2)]. There is substantial variation in the epidemiology of HIV infection by geographic region, with adult prevalence highest in sub-Saharan Africa at 5.2% in 2008. HIV prevalence has been falling in many countries over the last decade. Antiretroviral therapy (ART) substantially prolongs survival among people with HIV, and its wider availability is therefore expected to result in increased HIV prevalence. As a result the continuity of care for older persons living with HIV is crucial.

People living with HIV are at greater risk of developing noncommunicable diseases (more risk of developing cervical cancer, other cancers, mental illness, kidney and liver disease). It has been suggested that NCDs can be a direct consequence of HIV infection, increased behavioral and lifestyle risks and, in some cases, side effects of HAART regimes [[3](file:///H:\My%20Documents\My%20papers%20&amp;%20conferences\Submitted%20papers\Integration%20HIV%20and%20NCD\Paper\Revision%20June-Aug%202016\Supplementary%20file%202016.08.11.docx#_ENREF_3)].

Health services for HIV and noncommunicable diseases have common features since both require health systems that can provide for people’s chronic care needs [[3](file:///H:\My%20Documents\My%20papers%20&amp;%20conferences\Submitted%20papers\Integration%20HIV%20and%20NCD\Paper\Revision%20June-Aug%202016\Supplementary%20file%202016.08.11.docx#_ENREF_3)]. Therefore, long-term care for AIDS and NCDs present an opportunity to coordinate efforts and synergies between both programmes and their integration can be used to strengthen health systems. However, careful consideration is needed when deciding the approaches that are most cost-effective in the context of shortages of human resources and already overburdened health care professionals. Reliable data is needed on the burden of NCDs in HIV/AIDS-affected populations; the current and future burden of NCD co-morbidities in populations affected by HIV/AIDS; and evidence on the merits of the different models of integration.

Examples of integration include a study in Zambia offering HIV testing services to women who attend cervical cancer screening clinics, and visual screening for cervical cancer to women who attend HIV care and treatment clinics [[4](file:///H:\My%20Documents\My%20papers%20&amp;%20conferences\Submitted%20papers\Integration%20HIV%20and%20NCD\Paper\Revision%20June-Aug%202016\Supplementary%20file%202016.08.11.docx#_ENREF_4)]. Another example in Cambodia involves the introduction of single clinics for chronic diseases, where ART, diabetes, and hypertension have been successfully integrated [[5](file:///H:\My%20Documents\My%20papers%20&amp;%20conferences\Submitted%20papers\Integration%20HIV%20and%20NCD\Paper\Revision%20June-Aug%202016\Supplementary%20file%202016.08.11.docx#_ENREF_5)]. In Kenya, screening for blood pressure has been introduced in HIV programmes [[6](file:///H:\My%20Documents\My%20papers%20&amp;%20conferences\Submitted%20papers\Integration%20HIV%20and%20NCD\Paper\Revision%20June-Aug%202016\Supplementary%20file%202016.08.11.docx#_ENREF_6)] while in Ethiopia the tools and implementation strategies used to provide continuity of care for HIV are also being adopted to support diabetes services [[7](file:///H:\My%20Documents\My%20papers%20&amp;%20conferences\Submitted%20papers\Integration%20HIV%20and%20NCD\Paper\Revision%20June-Aug%202016\Supplementary%20file%202016.08.11.docx#_ENREF_7)]. Similarly, in Swaziland an existing HIV-specific site assessment tool has been adapted for diabetes and piloted in a rural health care facility [[8](file:///H:\My%20Documents\My%20papers%20&amp;%20conferences\Submitted%20papers\Integration%20HIV%20and%20NCD\Paper\Revision%20June-Aug%202016\Supplementary%20file%202016.08.11.docx#_ENREF_8)].

The aim of this review is to synthesise knowledge concerning the integrated delivery of NCDs and HIV services at health facility level. In addition, we will assess the experience of health care providers and patients and we will assess the macro and meso health system level barriers and facilitators to the integration of Non-Communicable Diseases and HIV programmes at service delivery level.

# Objectives

This systematic review has two complementary objectives. The first consists of synthesis knowledge concerning the existent models of NCDs and HIV integration at service delivery level. The second objective consists of analysing the health system barriers and facilitators to the integration of services.

## Objective 1 Models of integration

To synthesise knowledge concerning the integrated delivery of NCDs and HIV services at health facility level.

The specific objectives are:

• to summarise literature describing how NCDs and HIV services have been integrated, including both quantitative and qualitative reports; formal epidemiological studies and case studies; and without exclusions on the basis of lack of quantitative outcome data.

• to describe the strengths and weaknesses of the reported models of integration.

• to describe how effectiveness of integrated delivery has been measured.

• to identify gaps in the existing knowledge base and to formulate research priorities.

## Objective 2 Health System Barriers and Facilitators

To assess the evidence for health system level barriers and facilitators to the integration of NCDs and HIV programmes at service delivery level.

# Conceptual framework

## Definition of Integration

Based on the work of Groene, Briggs and Atun [[9-11](file:///H:\My%20Documents\My%20papers%20&amp;%20conferences\Submitted%20papers\Integration%20HIV%20and%20NCD\Paper\Revision%20June-Aug%202016\Supplementary%20file%202016.08.11.docx#_ENREF_9)] we defined integration as managerial or operational changes to health systems to bring together inputs, delivery, management and organisation of particular service functions as a means of improving access, quality, user satisfaction, equity and effectiveness. We considered this to include integrating ‘different packages of services’; the integration of service delivery points; integrating services to assure continuity of care; integration at different levels of service delivery; and integration of management decisions [[12](file:///H:\My%20Documents\My%20papers%20&amp;%20conferences\Submitted%20papers\Integration%20HIV%20and%20NCD\Paper\Revision%20June-Aug%202016\Supplementary%20file%202016.08.11.docx#_ENREF_12)]. We excluded integration of management decisions made at policy level since our focus was integration at service delivery level.

1. **Health systems barriers and facilitators**

Health systems have been defined by the WHO as “all the organizations, institutions and resources that are devoted to producing health actions”. A number of conceptual frameworks have been to illustrate the functioning of health systems. We will use the WHO health systems framework (figure 1) to classify any identified barriers or facilitators to effective integrated care according to the 6 health system building blocks.

Figure 1: WHO Health systems framework. (SOURCE WHO Western Pacific Region) <http://www.wpro.who.int/health_services/health_systems_framework/en/index.html>


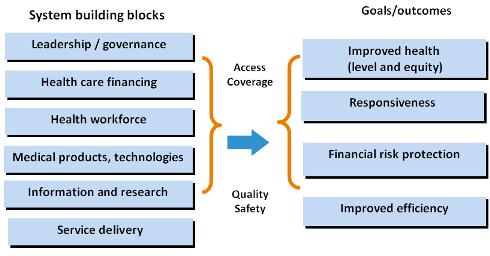


## Effects of interventions

When evaluating the effects of the interventions we will focus on measuring:

-Feasibility

-Effectiveness

-Quality of integrated services

-Coverage of integrated services

-Cost and Cost Effectiveness

-Other outcomes

# Methods

**Study eligibility**

## Type of chronic conditions

To be considered for inclusion the study will have to integrate a chronic disease with HIV. The list of chronic diseases included are Coronary Heart Diseases, Chronic Cardiovascular Diseases, Cerebrovascular Diseases (stroke), Hypertension, Diabetes Mellitus Type 2, Chronic Obstructive Pulmonary Disease, Asthma, Arthritis (Osteoarthritis, Rheumatoid Arthritis, Sacroiliitis, Spondylarthritis, Spondylarthropathies), Alzheimer Disease, Vascular Dementia, Depressive Disorders, Breast cancer, Cervical cancer, Colorectal cancer, Prostatic cancer and Lung cancer. We will also include studies that look at integration of mental health services or alcohol and substance misuse and HIV services. Papers looking at integration of HIV and Hepatitis C services will also be eligible.

**Inclusion criteria**

Reports will be included if they are a description or evaluation of a management or organisational change strategy, made within an existing health system, aiming to increase integration between HIV and NCDs care at service delivery level. Services could be provided in health facilities or in the community. Reports will have to describe experience of integration, not a theoretical account of how integration might be implemented. We will not exclude reports based on study design; nor will we require them to include outcome measures.

- A description or evaluation of a management or organisational change strategy, made within an existing health system, aiming to increase integration between HIV and Chronic conditions care at service delivery level.
- Reports will have to describe experience of integration, not a theoretical account of how integration might be implemented.
- Screening or treatment for HIV in a chronic disease treatment service and screening or treatment for chronic disease in a HIV treatment service will be included.
- Hepatitis C will be included as a chronic condition.
- Services could be provided in health facilities or in the community.
- We will not exclude reports based on study design; nor will we require them to include outcome measures.

**Study eligibility**

All studies that describe or evaluate the management or organisational change strategy, made within an existing health system, aiming to increase integration between HIV and Chronic conditions care at service delivery level.

**Eligibility criteria**

Population: Any population at the community, primary, secondary or tertiary care level will be included.

**Studies**

Any qualitative or quantitative study.

**Exclusion criteria**

To ensure a comprehensive descriptive review, we will not exclude studies based on their design, or absence of outcome measures. We will not restrict by ‘quality measures’ but will exclude ‘fatally flawed’ studies.

Health promotion or disease prevention papers will be excluded even if this activity is delivered in an HIV or chronic disease treatment setting.

Papers looking exclusively at integration of TB and HIV services will be excluded as they have been described in a previous recent review.

## Search strategy

The search strategy was developed to be consistent with methods used by other authors for systematic reviews of integration of health services [[10](file:///H:\My%20Documents\My%20papers%20&amp;%20conferences\Submitted%20papers\Integration%20HIV%20and%20NCD\Paper\Revision%20June-Aug%202016\Supplementary%20file%202016.08.11.docx#_ENREF_10), [13](file:///H:\My%20Documents\My%20papers%20&amp;%20conferences\Submitted%20papers\Integration%20HIV%20and%20NCD\Paper\Revision%20June-Aug%202016\Supplementary%20file%202016.08.11.docx#_ENREF_13)], and is described in detail in Appendix 3. We will search 15 bibliographic databases from inception to October 2012 including Medline, Embase and the Cochrane library; and abstracts from the International AIDS Society (IAS) Online Resource Library from 2006 to 2012, the HIV Implementers meetings from 2007 to 2012 (<http://www.hivimplementers.com>) and conferences on NCDs. We conducted hand-searching and consulted expert reviewers to check the completeness of the electronic searches and included additional papers as appropriate.

## Search and retrieval of studies

Two reviewers will independently go through the list of articles from the electronic database search results to identify those articles relevant to this systematic review based on title or title and abstract. If either of the two reviewers considered a study potentially eligible, the full article of this study will be retrieved for further assessment. The retrieved full texts will be assessed independently by the two reviewers to assess whether they meet the inclusion criteria for this review. Any disagreements concerning studies to be included will be resolved by consensus or by discussion with a third reviewer.

The eligibility criteria do not have language restrictions, if articles in languages other than English are identified inclusion will be assessed with the help of someone who can read and understand the articles. Further, no studies will be excluded based on assessment of bias.

Further potential studies will be identified by screening the reference lists of included articles.

## Data extraction

Two reviewers will independently extract data from included studies using standardized forms. We developed forms for extracting data on study characteristics and results for both quantitative and qualitative studies. These forms will be piloted by the reviewers on two randomly selected included studies and edited based on feedback received.

## Quality appraisal of quantitative studies

## Studies which present evaluative and not purely descriptive data, they will be independently assessed for risk of bias by two reviewers. For observational study designs, risk of bias will be assessed using a simple proforma for three domains: selection bias, information bias (differential misclassification & non-differential misclassification) and confounding. (*Appendix* 1) Risk of bias for each domain will be assessed as either low, unclear or high. Studies which have a low risk of bias in all domains will be classified as having a low overall risk of bias. For randomized studies the Cochrane risk of bias tool will be used.

## Quality appraisal of qualitative studies

Qualitative studies will be evaluated using an adapted version of a checklist used in a previous series of mixed methods systematic reviews incorporating both quantitative and qualitative studies [[14](file:///H:\My%20Documents\My%20papers%20&amp;%20conferences\Submitted%20papers\Integration%20HIV%20and%20NCD\Paper\Revision%20June-Aug%202016\Supplementary%20file%202016.08.11.docx#_ENREF_14), [15](file:///H:\My%20Documents\My%20papers%20&amp;%20conferences\Submitted%20papers\Integration%20HIV%20and%20NCD\Paper\Revision%20June-Aug%202016\Supplementary%20file%202016.08.11.docx#_ENREF_15)] (Appendix 2). The checklist used here will add a further three quality criteria to the seven criteria used in Rees et al.[[14](file:///H:\My%20Documents\My%20papers%20&amp;%20conferences\Submitted%20papers\Integration%20HIV%20and%20NCD\Paper\Revision%20June-Aug%202016\Supplementary%20file%202016.08.11.docx#_ENREF_14)] These additional three criteria relate to: the generalisabilty of the study, consideration of the impact of the researcher’s views and interactions on the study (reflexivity), and consideration of ethical issues. These additional criteria are found in a number of published tools for quality appraisal of qualitative research. [[16-19](file:///H:\My%20Documents\My%20papers%20&amp;%20conferences\Submitted%20papers\Integration%20HIV%20and%20NCD\Paper\Revision%20June-Aug%202016\Supplementary%20file%202016.08.11.docx#_ENREF_16)]

## Presentation and synthesis of results

Qualitative and quantitative studies results will be presented separately.

Where possible we will report dichotomous outcomes as relative risks (RR) and continuous data as mean differences (MD) (with 95% confidence intervals). Data will also be presented with an indication of whether the intervention had a positive effect (+), a negative effect (-), or no statistically significant effect (0).

Studies will be coded within the different models identified (Objective 1), and according to the 6 building blocks of the WHO health systems framework (Objective 2) Due to expected substantial heterogeneity in study design, exposures, participants and outcomes, and the nature of this systematic review question we do not plan to pool studies in a meta-analysis but instead present a configurative synthesis of the findings [[20](file:///H:\My%20Documents\My%20papers%20&amp;%20conferences\Submitted%20papers\Integration%20HIV%20and%20NCD\Paper\Revision%20June-Aug%202016\Supplementary%20file%202016.08.11.docx#_ENREF_20)]. Organizing the findings into the different frameworks will be conducted independently by two reviewers and disagreement will be discussed. Although it is expected that all findings identified in the literature will fit into one of the domains proposed by our proposed framework if new dimensions are identified they will be added and the conceptual framework refined and updated accordingly. The frequency of studies reporting the assessment of each domain will be presented. This research mapping will inform gaps in the current literature and will help to identify areas that require further investigation.

If data allows we plan to conduct subgroup analyses according to geographical regions, national income, and study setting (urban versus rural).

# References

1. WHO, *Noncommunicable Diseases Country Profiles 2011*. 2011, World Health Organization: Geneva, Switzerland.

2. UNAIDS, *AIDS epidemic update: November 2009*. 2009. [Accessed 8th August 2010]. Available from: <http://data.unaids.org/pub/Report/2009/JC1700_Epi_Update_2009_en.pdf>, UNAIDS and World Health Organization: Geneva.

3. UNAIDS, *Chronic care of HIV and noncommunicable diseases*. 2011: Geneva.

4. Pfaendler, K., M. Mwanahamuntu, and V. Sahasrabuddhe, *Management of cryotherapy-ineligible women in a “screen-and-treat” cervical cancer prevention program targeting HIV-infected women in Zambia: lessons from the field.* Gynecol Oncol, 2008. **110**: p. 402-407.

5. Janssens, B., et al., *Offering integrated care for HIV/AIDS, diabetes and hypertension within chronic disease clinics in Cambodia.* Bulletin of the World Health Organization, 2007. **85**(11): p. 821-900.

6. Mwangemi, F., P. Lamptey, and G. Yonga, *Health Systems Strengthening: CVD/HIV Integration Initiative, FHI/Kenya Case Study*, in *Diabetes Leadership Forum*. 2010.

7. Melaku, Z., A. Reja, and M. Rabkin, *Strengthening Health Systems for Chronic Care and NCDs: Leveraging HIV Programs to Support Diabetes Services in Ethiopia*, in *IAS 2011*. 2011: Rome, Italy.

8. Rabkin, M., et al., *Strengthening Health Systems for Chronic Care: Leveraging HIV Programs to Support Diabetes Services in Ethiopia and Swaziland*.

9. Groene, O. and M. Garcia-Barbero, *Integrated care: a position paper of the WHO European office for integrated health care services.* International Journal of Integrated Care, 2001. **1**: p. 1-10.

10. Briggs, C. and P. Garner, *Strategies for integrating primary health services in middle- and low-income countries at the point of delivery.* Cochrane Database of Systematic Reviews, 2006. **2**: p. CD003318.

11. Atun, R., et al., *Integration of targeted health interventions into health systems: a conceptual framework for analysis.* Health Policy and Planning, 2010. **25**: p. 104-111.

12. WHO. *Integrated health services – what and why? Technical Brief No. 1*. 2008. [Accessed 20^th^ May 2010]. Available from: <http://www.who.int/healthsystems/technical_brief_final.pdf>.

13. Atun, R., T. de Jongh, and F. Secci, *A systematic review of the evidence on integration of targeted health interventions into health systems.* Health Policy and Planning, 2010. **25**: p. 1-14.

14. Rees, R., et al., *Young People and Physical Activity: A systematic review of barriers and facilitators*. 2001, EPPI-Centre, Social Science Research Unit, Institute of Education, University of London: London.

15. Harden, A., et al., *Young People and Mental Health: A Systematic Review of Research on Barriers and Facilitators.* 2001, EPPI-Centre, Social Science Research Unit: London.

16. Mays, N. and C. Pope, *Rigour and qualitative research.* BMJ., 1995. **311**(6997): p. 109-12.

17. Boulton M, Fitzpatrick R, and S. C., *Qualitative research in health care: a structured review and evaluation of studies.* J Eval Clin Pract 1996. **2**: p. 171-9.

18. British Sociology Association Medical Sociology Group, *Criteria for the evaluation of qualitative research papers.* Medical Sociology News, 1996. **22**(1): p. 69-71.

19. Critical Appraisal Skills Programme, *10 questions to help you make sense of qualitative research*. 2010.

20. Gough, D., S. Oliver, and J. Thomas, *An Introduction to Systematic Reviews*. 2012, London: Sage Publications.

Appendix 1 – Tool for assessing risk of bias for observational studies

| **Type of bias** | **Study design** | | | |
| --- | --- | --- | --- | --- |
|  | **Cross sectional** | **Case control** | **Cohort** | **Ecological** |
| Selection bias | Was the study population selected appropriate? | | | |
|  | Was the sample representative of its target population? | Were the controls randomly selected from the same population as the cases? | Was an appropriate control group used?  Was follow up sufficiently complete? (>80%) | Were the subjects representative of the group, place, or population of interest? |
| Differential misclassification | Did the assessment of the exposure or outcome differ according to the patient status? | Did the exposure assessment differ for cases and controls? | Did the outcome assessment differ for exposed and non exposed? | Were the exposure and outcome variables measured and defined in the same or a similar way across the different groups studied? |
| Non-differential misclassification | Were valid methods used for measuring hypertension awareness, treatment or control and medication adherence? | | | |
| Confounding | Was any strategy undertaken to control for potential confounders?   1. At the design stage (restriction, matching) 2. At the analysis stage (stratification, multivariable analysis) | | | |

**Define each domain as low risk of bias, unclear risk of bias or high risk of bias**

.

# Appendix 2: Tool for quality appraisal of qualitative studies

| Domain | Criteria for assessment | Yes or No |
| --- | --- | --- |
| Background | Did the report provide an explanation and justification for the focus of the study and methods used?  Is there an explicit account of a supporting theoretical framework and/or a supporting literature review? |  |
| Aims and objectives of the study | Did the report explicitly and clearly state the aims of the study? |  |
| Context | Did the report adequately describe the specific circumstances under which the research was developed, carried out and completed? |  |
| Sampling | Did the report provide adequate details of the sampling strategy used, including methods for sampling and recruitment, and characteristics of subjects included in the study? |  |
| Description of data collection and analysis | Were both data collection and data analysis methods clearly described in the report? |  |
| Reliability of data analysis | Does the report provide evidence of attempts to demonstrate the reliability of data analysis? For example did more than one researcher check the coding or did the researchers present evidence that participant accounts had been faithfully represented? |  |
| Clarity about how conclusions were derived | Was sufficient original data - such as quotations, data tables or observations - included in the report to confirm the links made between data and interpretations or conclusions? |  |
| Reflexivity | Did the report reflect on the impact on the findings of the researchers own views and relationships with the participants? |  |
| Generalisability | Did the report outline the extent to which any findings can be generalised beyond the settings and participants of study and acknowledge the limits of generalisability? |  |
| Ethics | Was their evidence of consideration of ethical issues, such as confidentiality, in the report? |  |

# Appendix 3: Search strategy

**Search Strategy used for Medline, Embase and Global Health via Ovid**

| Database: Embase <1980 to October 2015>, Global Health <1910 to October 2015>, Ovid MEDLINE(R) <1946 to October Week 4 2015>  1. ((vertical or horizontal or integrat* or coordinat* or co-ordinat* or link*) and (program* or care or service*)).mp. or delivery of health care, integrated/ or primary healthcare/  2. exp HIV infections/ or HIV.mp. or Human immunodeficiency virus.mp. or "HIV/aids".mp.  3. (All introduced in a separate line) chronic disease/ or long-term care/ or ((chronic* or persistent or long* term or ongoing or degenerative) adj3 (disease* or disab* or ill* or condition* or health condition* or medical condition*)).tw. or long* term care.tw. or (non-communicable disease* or NCD).tw. or exp neurodegenerative diseases/ or (neurodegenerative or Huntington* disease or Parkinson* disease or amyotrophic lateral sclerosis or motor neuron disease).tw. or exp multiple sclerosis or multiple sclerosis.tw. or exp arthritis/ or (arthritis or osteoarthritis or rheumati*).tw. or exp lung diseases obstructive/ or (obstructive lung disease* or obstructive pulmonary disease* or asthma or bronchitis).tw. or exp emphysema/ or exp pulmonary emphysema/ or emphysema.tw. or exp diabetes mellitus/ or (diabetes or diabetic).tw. or exp hypertension/ or (hypertension or high blood pressure).tw. or exp cerebrovascular disorders/ or (cerebrovascular disease* or cerebrovascular disorder* or brain ischaemia or cerebral infarction or carotid artery disease* or stroke).tw. or exp dementia/ or (dementia or alzheimer*).tw. or exp epilepsy/ or epilep*.tw. or exp myocardial ischemia/ or (myocardial isch* or ischaemic heart disease or ischemic heart disease or angina or coronary disease* or coronary heart disease* or coronary artery disease* or myocardial infarction).tw. or exp heart failure/ or heart failure.tw. or exp renal insufficiency/ or ((renal or kidney) adj (failure* or insufficienc*)).tw. or exp colonic diseases/ or (colonic disease* or colitis or irritable bowel syndrome).tw. or exp obesity/ or (obesity or obese).tw. or exp osteoporosis/ or osteoporosis.tw. or fibromyalgia/ or fibromyalgia*.tw. or exp neoplasms/ or (cancer* or oncolog* or neoplasm* or carcinom* or tumo?r* or malignan*).tw. or exp cataract/ or (cataract or blindness or visual loss or visual impairment or loss of vision).tw. or exp hearing loss/ or exp deafness/ or deafness.mp. and hearing loss.tw. [mp=ti, ab, sh, hw, tn, ot, dm, mf, dv, kw, bt, nm, ps, rs, ui] or exp depression/ or exp mental disorder/ or (mental health or depression).tw. or exp alcoholism/ or alcohol*.tw. or exp substance-related disorders or substance misuse.tw.  4. 1 and 2 and 3 |
| --- |

1. Where N is the total number of papers found to refer to facilitators or barriers (respectively) associated with a particular block [↑](#footnote-ref-1)
